# Prevalence and Risk Factors of Urinary Tract Infection in Hospitalized Hip Fracture Patients: A Cross-Sectional Study

**DOI:** 10.7759/cureus.111485

**Published:** 2026-06-25

**Authors:** Karn Rojjananukulpong, Jirapong Leeyaphan, Piyapong Intarasompun, Yuthasak Peerakul

**Affiliations:** 1 Department of Disease Control, Bamrasnaradura Infectious Diseases Institute, Nonthaburi, THA

**Keywords:** estimated glomerular filtration rate (egfr), hip fractures, prevalence, risk factors, the osteoporosis self-assessment tool for asians (osta) index, urinary tract infections

## Abstract

Background

Urinary tract infection (UTI) is a common complication among hospitalized patients with hip fractures. Several risk factors may increase the likelihood of UTI in this population and can lead to serious complications. This cross-sectional study aims to determine the prevalence of UTI and identify associated risk factors among hospitalized hip fracture patients at a tertiary public hospital in Thailand.

Methods

We conducted a retrospective cross-sectional study of 140 hospitalized patients with hip fractures from April 2016 to March 2025 and analyzed the prevalence of UTIs and associated risk factors. Logistic regression analysis was used to assess the odds ratio (OR) and 95% confidence intervals (CI).

Results

UTI occurred in 51 participants (36.4%) of hospitalized hip fracture patients. The mean age in the UTI group was 81.4 years, and UTI was more common in females. Female sex (OR 5.109; 95% CI 1.164 - 22.426), the Osteoporosis Self-Assessment Tool for Asians (OSTA) index ≤ -6 (OR 5.798; 95% CI 1.343 - 25.032), and the estimated glomerular filtration rate (eGFR) stage ≥ 2 (OR 8.946; 95% CI 1.477 - 54.178) were significant risk factors.

Conclusion

UTI was common among hospitalized hip fracture patients. Female sex, OSTA ≤ -6, and eGFR stage ≥ 2 were the main associated risk factors.

## Introduction

Geriatric hip fractures are a major age-related health burden worldwide. Their prevalence is increasing in many countries because of population aging [1]. Although surgical treatment and multidisciplinary care have improved outcomes, complications during hospitalization remain common. These include pneumonia, urinary tract infection (UTI), and pressure ulcers, which may result from both preventable and non-preventable factors. Such complications can adversely affect outcomes, including length of stay, hospital costs, and ambulatory status [2,3].

UTI is a common complication in older adults with hip fractures. An active UTI not only prolongs hospital recovery and delays rehabilitation, but they also serve as a primary source for hematogenous seeding, elevating the risk of periprosthetic joint infection [4]. Previous studies reported a hospitalization-related UTI prevalence ranging from 8% to 52% in this population [5-8]. In Thailand, data on UTI prevalence among hospitalized older adults with hip fractures remain limited. One Thai study reported a prevalence of 28.32% [9]. This wide variation suggests that multiple factors may influence UTI risk. Identifying these factors may help predict UTI, improve care processes, and reduce its incidence, ultimately leading to better outcomes [10].

Previous studies have identified several risk factors for UTI in patients with hip fractures, including patient-related factors: age over 85 years, female sex, obesity, intertrochanteric fracture, delirium, and prolonged hospital stay. Comorbidity-related factors: the American Society of Anesthesiologists Classification (ASA) class ≥ 3, dementia, Parkinson's disease, diabetes, hypertension, congestive heart failure, sepsis, and a history of chronic steroid use. Procedure-related factors: catheterization, blood transfusion, general anesthesia, hemiarthroplasty, and surgery delayed more than two days after admission [11-13]. However, few studies in Thailand have examined UTI risk factors in hip fracture patients throughout hospitalization. Additionally, some independent risk factors for UTI in the general population were not evaluated. The objectives of this study were twofold: 1) to estimate the overall prevalence of UTIs among patients hospitalized with hip fractures, and 2) to identify independent risk factors associated with the development of these infections.

## Materials and methods

Study population

We retrospectively reviewed the medical records of hospitalized patients with hip fractures at a tertiary public hospital from April 2016 to March 2025. Patients with femoral neck, intertrochanteric, or subtrochanteric fractures who had urinalysis results were included. Patients with hip fractures due to primary or metastatic carcinoma were excluded. The study was approved by the Institutional Review Board (S015h/67_ExPD).

Data collection and measurement

Data were extracted from medical records into case record forms by all authors, including age, sex, weight, height, fracture type, comorbidities (diabetes, hypertension, dyslipidemia), smoking, alcohol use, steroid use, previous fracture, previous UTI, previous hospitalization, American Society of Anesthesiologists (ASA) classification, anesthesia type, time from admission to surgery, length of stay, hemoglobin, creatinine, and urinalysis results.

UTI information was obtained from the discharge summaries of the medical records; patients diagnosed with a UTI but without urinalysis results were excluded.

The OSTA index was calculated by the formula of 0.2 × (body weight - age), in which the decimals were truncated to an integer [14]. Estimated glomerular filtration rate (eGFR) was calculated by the Chronic Kidney Disease Epidemiology Collaboration (CKD-EPI) equation [15]. Hemoglobin level was used to diagnose anemia according to the World Health Organization criteria. Anemia in women was defined as a hemoglobin concentration less than 12 g/dL. Anemia in men was defined as a hemoglobin concentration less than 13 g/dL [16].

Statistical analysis

Participants were grouped by UTI status. Baseline characteristics were summarized as mean ± standard deviation or median with interquartile range, as appropriate for data distribution. Group comparisons were performed using chi-squared test, Student's t-test, analysis of variance (ANOVA), or Mann-Whitney U test, as appropriate. Binary logistic regression was used to assess factors associated with UTI. Variables for the multivariable model were selected based on bivariate significance and clinical relevance, and backward stepwise logistic regression was used to refine the final model. Based on an expected UTI prevalence of 28% [9] and a margin of error of 7.5%, the required sample size was 138. Statistical analyses were performed using STATA version 15 (StataCorp LLC, College Station, TX, USA), and p < 0.05 was considered statistically significant.

## Results

**Figure 1:**
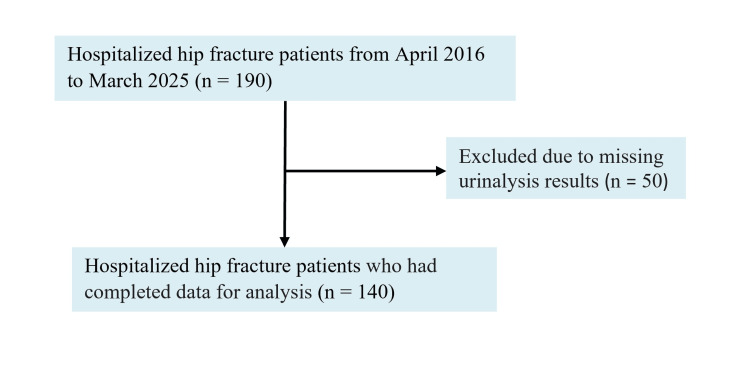
Flow of subject enrollment

A total of 190 hospitalized patients with hip fractures were screened. Because urinalysis was not performed routinely but rather indicated by suspected UTI. As a result, fifty were excluded because urinalysis results were unavailable, and none had hip fractures due to primary or metastatic carcinoma. The final analysis included 140 patients (Figure 1).

Demographic and prevalence of UTI

The prevalence of UTI was 51 participants (36.4%). Baseline characteristics are presented in Table 1. Patients with UTI were older than those without UTI (mean age 81.4 years; p < 0.001), and UTI was more common in females (p < 0.001). Weight, height, body mass index (BMI), Osteoporosis Self-Assessment Tool for Asians (OSTA) index, the estimated glomerular filtration rate (eGFR), ASA classification, and history of UTI differed significantly between the groups (all p < 0.05) as shown in Table 2 and Table 3.

**Table 1:** Clinical characteristics of patients Variables are presented as mean ± standard deviation or n (%). UTI - urinary tract infection; BMI - body mass index; OSTA - Osteoporosis Self-Assessment Tool for Asians; LOS - length of stay; eGFR - estimated glomerular filtration rate; ASA - American Society of Anesthesiologists Classification

Variables	Total (n= 140)
Age (years)	75.7 ± 13.0
Sex	
Female	111 (79.3)
Male	29 (20.7)
Weight (kg)	50.9 ± 11.3
Height (cm)	155.3 ± 8.9
BMI (kg/m^2^)	21.1 ± 4.1
OSTA index	−4.7 ± 3.8
LOS (day)	14.7 ± 9.9
Time from admission to surgery (hour)	90.4 ± 57.1
eGFR (ml/min/1.73 m^2^)	67.1 ± 27.3
Hemoglobin (g/dL)	10.9 ± 1.8
UTI	51 (36.4)
Fracture type	
Femoral neck	79 (56.4)
Intertrochanteric	55 (39.3)
Subtrochanteric	6 (4.3)
Treatment	
Operation	131 (93.6)
Conservative	9 (6.4)
Operative type	
Arthroplasty	70 (50.0)
Nail	44 (31.4)
Dynamic hip screw	17 (12.1)
ASA Class (n= 131)	
2	13 (9.3)
3	117 (83.6)
4	1 (0.7)
Anesthetic type (n= 131)	
General anesthesia	79 (56.4)
Regional anesthesia	52 (37.1)
Diabetes mellitus	49 (35.0)
Hypertension	99 (70.7)
Dyslipidemia	73 (52.1)
Previous history of UTI	27 (19.3)
Previous history of hospital admission	45 (32.1)
Previous history of fracture	22 (15.7)
Alcohol	1 (0.7)
Smoking	1 (0.7)
Steroid usage	1 (0.7)

**Table 2:** Comparison of participant demographics between the UTI and non-UTI groups Variables are presented as mean ± standard deviation UTI - urinary tract infection; BMI - body mass index; OSTA - Osteoporosis Self-Assessment Tool for Asians; LOS - length of stay; eGFR - estimated glomerular filtration rate

Variables	UTI group (n = 51)	Non-UTI group (n= 89)	Mean difference	t-value	p-value
Age (years)	81.4 ± 7.6	72.4 ± 14.3	8.991	4.156	<0.001
Weight (kg)	46.1 ± 9.5	53.6 ± 11.4	−7.520	−3.985	<0.001
Height (cm)	153.0 ± 9.2	156.6 ± 8.5	−3.663	−2.380	0.019
BMI (kg/m^2^)	19.7 ± 3.7	21.9 ± 4.1	−2.145	−3.098	0.002
OSTA index	−6.6 ± 2.6	−3.5 ± 3.9	−3.080	−5.014	<0.001
LOS (day)	17.2 ± 13.3	13.3 ± 6.9	3.820	1.913	0.060
Time from admission to surgery (hours)	99.4 ± 63.1	85.7 ± 53.5	13.672	1.238	0.219
eGFR (ml/min/1.73 m^2^)	59.7 ± 25.4	71.4 ± 27.6	−11.675	−2.477	0.014
Hemoglobin (g/dL)	10.8 ± 1.8	11.0 ± 1.9	−0.203	−0.634	0.532

**Table 3:** Comparison of participant demographics between the UTI and non-UTI groups Variables are presented as n (%). UTI - urinary tract infection; BMI - body mass index; ASA - American Society of Anesthesiologists Classification * Chi-squared test was used for comparisons; ** Fisher's exact test was used because one or more expected cell counts were less than 5.

Variables	UTI group, (n, %)	Non-UTI group, (n, %)	Chi-squared value (ꭓ^2^)	p-value
Sex*			5.815	0.016
Female	46 (90.2)	65 (73.0)		
Male	5 (9.8)	24 (27.0)		
Fracture type*			0.028	0.986
Femoral neck	29 (56.9)	50 (56.2)		
Intertrochanteric	20 (39.2)	35 (39.3)		
Subtrochanteric	2 (3.9)	4 (4.5)		
Treatment**			-	0.073
Operation	45 (88.2)	86 (96.6)		
Conservative	6 (11.8)	3 (3.4)		
Operative type*			2.943	0.230
Arthroplasty	24 (53.3)	46 (53.5)		
Nail	18 (40.0)	26 (30.2)		
Dynamic hip screw	3 (6.7)	14 (16.3)		
ASA Class*			8.202	0.017
2	0 (0)	13 (15.1)		
3	45 (100.0)	72 (83.7)		
4	0 (0)	1 (1.2)		
Anesthetic type*			0.646	0.422
General anesthesia	25 (55.6)	54 (62.8)		
Regional anesthesia	20 (44.4)	32 (37.2)		
Diabetes mellitus*			0.003	0.956
Yes	18 (35.3)	31 (34.8)		
No	33 (64.7)	58 (65.2)		
Hypertension*			0.001	0.980
Yes	36 (70.6)	63 (70.8)		
No	15 (29.4)	26 (29.2)		
Dyslipidemia*			0.043	0.835
Yes	26 (51.0)	47 (52.8)		
No	25 (49.0)	42 (47.2)		
Previous history of UTI*			5.285	0.022
Yes	15 (29.4)	12 (13.5)		
No	36 (70.6)	77 (86.5)		
Previous history of hospital admission*			1.840	0.175
Yes	20 (39.2)	25 (28.1)		
No	31 (60.8)	64 (71.9)		
Previous history of fracture*			0.226	0.634
Yes	9 (17.6)	13 (14.6)		
No	42 (82.4)	76 (85.4)		
Alcohol**			-	1.000
Yes	0 (0)	1 (1.1)		
No	51 (100)	88 (98.9)		
Smoking**			-	1.000
Yes	0 (0)	1 (1.1)		
No	51 (100)	88 (98.9)		
Steroid usage**			-	0.364
Yes	1 (2.0)	0 (0)		
No	50 (98.0)	89 (100)		

Risk factors for UTI

Risk factors were grouped based on statistical cutoffs or previous reports [12,17]. The categories were age ≥ 80 years, weight ≤ 46 kg, height < 150 cm, BMI < 18 kg/m2, OSTA ≤ -6, LOS > 14 days, time from admission to surgery ≥ 72 hours, and eGFR stage ≥ 2. Fracture type was classified as intertrochanteric fracture versus other fracture types. Surgery type was classified as arthroplasty versus non-arthroplasty. Treatment was classified as operative versus nonoperative.

In univariable logistic regression, age ≥ 80 years, female sex, weight ≤ 46 kg, BMI ≤ 18 kg/m2, OSTA ≤ -6, eGFR stage ≥ 2, and a history of UTI were significantly associated with UTI (all p < 0.05) (Table 4). Length of stay > 14 days, time from admission to surgery ≥ 72 hours, fracture type, operative type, anesthetic type, diabetes mellitus, and hypertension were also included in the model because of their clinical relevance [8]. In both the multivariable and backward stepwise logistic regression models, female sex, OSTA ≤ -6, and eGFR stage ≥ 2 remained significant risk factors (p = 0.031, 0.019, and 0.017, respectively). However, the pseudo-R-squared of the multivariable model was higher than the backward stepwise logistic regression model (pseudo-R-squared = 0.2529 and 0.1709, respectively) as shown in Table 5 and Table 6.

**Table 4:** Univariable logistic regression analysis of risk factors OR - odds ratio; CI - confidence interval; BMI - body mass index; OSTA - Osteoporosis Self-Assessment Tool for Asians; LOS - length of stay; eGFR - estimated glomerular filtration rate; UTI - urinary tract infection

Variables	OR	95% CI	p-value
Age ≥ 80 years	2.545	1.256 - 5.155	0.010
Female	3.397	1.207 - 9.561	0.021
Weight ≤ 46 kg	2.583	1.268 - 5.264	0.009
Height ≤ 150 cm	1.886	0.925 - 3.848	0.081
BMI ≤ 18 kg/m^2^	2.679	1.185 - 6.053	0.018
OSTA ≤ −6	4.966	2.351 -10.490	<0.001
LOS > 14 days	1.198	0.591 - 2.430	0.616
Time from admission to surgery ≥ 72 hours	0.636	0.305 - 1.328	0.228
eGFR stage ≥ 2	2.497	1.173 - 5.313	0.018
Anemia	1.486	0.690 - 3.204	0.312
Fracture type	0.973	0.486 - 1.948	0.937
Operation	0.262	0.062 - 1.096	0.066
Operative type	0.994	0.482 - 2.048	0.987
Anesthetic type	0.741	0.356 - 1.541	0.422
Diabetes mellitus	1.021	0.496 - 2.099	0.060
Hypertension	0.990	0.465 - 2.109	0.980
Dyslipidemia	0.929	0.467 - 1.851	0.835
Previous history of UTI	2.674	1.136 - 6.293	0.024
Previous history of hospital admission	1.652	0.798 - 3.420	0.177
Previous history of fracture	1.253	0.494 - 3.174	0.635

**Table 5:** Multivariable logistic regression analysis of risk factors Pseudo-R squared = 0.2529 OR - odds ratio; CI - confidence interval; BMI - body mass index; OSTA - Osteoporosis Self-Assessment Tool for Asians; LOS - length of stay; eGFR - estimated glomerular filtration rate; UTI - urinary tract infection

Variables	OR	95% CI	p-value
Age ≥ 80 years	0.712	0.224 - 2.267	0.566
Female	5.109	1.164 - 22.426	0.031
Weight ≤ 46 kg	0.473	0.126 - 1.773	0.267
BMI ≤ 18 kg/m^2^	1.413	0.371 - 5.386	0.613
OSTA ≤ −6	5.798	1.343 - 25.032	0.019
LOS > 14 days	0.454	0.171 - 1.205	0.113
Time from admission to surgery ≥ 72 hours	0.548	0.218 - 1.379	0.202
eGFR stage ≥ 2	8.946	1.477 - 54.178	0.017
Fracture type	0.080	0.005 - 1.237	0.071
Operative type	0.089	0.006 -1.343	0.081
Anesthetic type	0.699	0.265 - 1.846	0.470
Diabetes mellitus	1.236	0.455 - 3.358	0.678
Hypertension	0.470	0.151 - 1.470	0.194
Previous history of UTI	2.021	0.669 - 6.109	0.212

**Table 6:** Backward stepwise logistic regression analysis of risk factors Pseudo-R squared = 0.1709 OR - odds ratio; CI - confidence interval; OSTA - Osteoporosis Self-Assessment Tool for Asians; eGFR - estimated glomerular filtration rate

Variables	OR	95% CI	p-value
Female	3.804	1.007 - 14.369	0.049
OSTA ≤ −6	3.151	1.381 - 7.190	0.006
eGFR stage ≥ 2	6.126	1.310 - 28.653	0.021

## Discussion

The reported prevalence of UTI among hospitalized patients with hip fractures varies across studies, likely because of differences in study populations, clinical settings, and diagnostic criteria. In the present study, the prevalence was 36.4%, which was higher than that reported in a previous Thai study [9]. Female sex, OSTA ≤ -6, and eGFR stage ≥ 2 were identified as potential risk factors for UTI in this population.

Previous studies have reported a UTI prevalence of 8% to 52% among older adults hospitalized with hip fractures [5-8]. The prevalence in the present study was higher than that in a prior Thai study (36.4% vs. 28.3%) [9]. Female sex was also a significant risk factor, consistent with patterns seen in the general population [18].

Renal function was also associated with UTI in this study. Because eGFR is estimated from serum creatinine and age [15], it should be interpreted cautiously in older adults with hip fractures. Previous studies have identified impaired renal function as a potential risk factor for UTI [19]. Patients with abnormal renal function may therefore require closer monitoring during hospitalization.

A previous study reported a relationship between bone mineral density and the risk of common infections and sepsis [20]. This study also identified OSTA ≤ -6 as a potential risk factor for UTI. The OSTA index is calculated as 0.2 × (body weight − age), with the decimal truncated to an integer [14], and lower scores generally reflect older age and lower body weight. Thus, older patients with lower body weight may be at greater risk of UTI. Moreover, this suggests that the OSTA index, which integrates age and body weight, is a more sensitive indicator of physical frailty and low physiologic reserve than age alone in this population. Low body weight and advanced age collectively reflect nutritional depletion and potential immune vulnerability, explaining why the combined index outperformed chronological age in predicting infection. This present study uses the OSTA index, which was originally developed for osteoporosis screening rather than infection prediction. Applying it as a risk factor for UTI is novel. Future studies might directly elucidate the relationship between bone mineral density and UTI. Our findings indicate that a low BMI increases a patient's risk of developing a UTI. This finding differs from previous reports that identified obesity as an independent risk factor for UTI [12]. Larger studies with subgroup analyses of body weight and nutritional status are needed to clarify the relationship between body composition and UTI risk in patients with hip fractures.

Limitations

The primary strengths of this study include the evaluation of a relatively long period and the implementation of multivariable analysis to rigorously control for potential confounders. However, this study has several limitations. First, it was conducted at a single center, which may limit generalizability. Second, because the present study was a retrospective study, some potentially important UTI-related factors reported in previous studies, such as the use and duration of urinary catheterization and microbiology results, were not assessed. Third, the clinical consequences of UTI, including patient outcomes and hospital costs, were not evaluated. Future studies should include larger multicenter cohorts and examine both short-term and post-discharge consequences, including recurrence. Finally, excluding 26% of patients may have introduced selection bias. Although the study evaluates risk factors previously identified in the literature, including multiple factors in the full model may weaken the reliability of the reported associations because of potential overfitting.

## Conclusions

Female sex, low eGFR, and lower OSTA scores were associated with UTI during hospitalization for hip fracture. Although these factors may not be modifiable at admission, early identification may help healthcare teams target other preventable contributors to UTI and improve patient care and outcomes.
